# Video Laryngoscopy Improves the Success of Neonatal Tracheal Intubation for Novices but Not for Experienced Medical Staff

**DOI:** 10.3389/fped.2020.00445

**Published:** 2020-08-06

**Authors:** Ming Zhou, Xiaohong Xi, Min Li, Silu Wang, Zhiqiang Liu, Jiang-Qin Liu

**Affiliations:** ^1^Department of Neonatology, Shanghai First Maternity and Infant Hospital, Tongji University School of Medicine, Shanghai, China; ^2^Department of Anesthesiology, Shanghai First Maternity and Infant Hospital, Tongji University School of Medicine, Shanghai, China

**Keywords:** simulation, neonate, tracheal intubation, video laryngoscopy, resuscitation

## Abstract

**Background:** There is limited evidence on the use of video laryngoscopy (VL) in neonatal tracheal intubation (NTI) during neonatal resuscitation. In this study, we aimed to compare the difference between direct laryngoscopy (DL) and VL in NTI of trainees during neonatal resuscitation training.

**Materials and Methods:** A prospective observational study was conducted during a neonatal resuscitation training course to examine three circumstances: NTI by experienced medical staff (EMS) and less-experienced medical staff (LEMS) in a neonatal resuscitation scenario; NTI by EMS and LEMS with an ongoing chest compression; and NTI by midwives who were novices in the procedure. The trainees were given scenarios or were shown demonstrations on newborn simulation manikins and were required to perform an NTI on a simulation manikin using DL and/or VL. The mean intubation time and success rate of intubation were measured.

**Results:** The mean NTI time for EMS using VL (24.1 ± 7.2 s) was significantly longer than that using DL intubation (18.1 ± 6.9 s, *P* < 0.001), whereas there was no significant difference between using VL and DL for LEMS. EMS spent slightly less time on NTI than did LEMS using both VL and DL, but there were no statistically significant differences (both *p* > 0.05). The NTI success rate for EMS using VL (48.0%, 12/25) was significantly lower than that using DL (88.0%, 22/25, *P* = 0.004), while the NTI success rate for LEMS using VL (68.2%, 15/22 vs. 40.9%, 9/22) was higher than that using DL, but there was no statistical significance. When NTI was required with ongoing chest compressions, there was no significant difference in the mean NTI time and success rate between using VL and DL for EMS or LEMS. In the group of midwives who were novices in NTI, after they watched a demonstration teaching NTI, the intubation time using VL (19.6 ± 9.0 s) was significantly shorter than that using DL (28.0 ± 6.7 s, *P* < 0.001). The success rate of NTI using VL was significantly higher (96.2%; 25/26) than that using DL (69.2%; 18/26).

**Conclusion:** The video laryngoscopy could be an effective training tool for inexperienced staff in developing the skill of tracheal intubation.

## Background

Intrapartum-related complications or birth asphyxia is one of the leading causes of neonatal death worldwide and in China (1). Worldwide, 0.7 million newborns die of birth asphyxia, accounting for 9% of childhood deaths under 5 years of age (2). It is also a major cause of neonatal morbidity and long-term sequelae (3). Some of the neonatal deaths associated with birth asphyxia can be prevented by prompt and effective neonatal resuscitation (4). Therefore, the American Academy of Pediatrics and the Chinese Academy of Pediatrics have recommended that at least one well-trained resuscitator should be present during delivery to address the possible risks (5, 6). Every medical staff member in the delivery room must be trained in neonatal resuscitation skills, including the initial step of resuscitation, positive pressure ventilation, intubation, chest compression, and medication administration via an umbilical vein catheters (UVC), in case neonatal resuscitation is necessary.

Among those who need resuscitation right after birth in the delivery room, ~3% of newborns need some help to start breathing, and 2% require tracheal intubation (TI) for further resuscitation (7). TI is an invasive and critical procedure. It is challenging for a resuscitator to intubate an ill newborn within a limited duration, mostly within 30 s, under the high stress associated with highly adverse events (8). It has been investigated whether medical staff has had decreasing opportunities for the practice of TI in the NICU (9). On the other hand, in 2015, NRP2015 recommended that TI should be performed to establish an artificial airway when external chest heart massage is performed. When CPR is started, it is usually necessary to stop massage to reduce the risk of TI failure (10). In recent years, video laryngoscopy (VL) has been widely used in adult and pediatric patients to establish artificial airways (11, 12). VL improves the view of the glottis and might reduce the risk of adverse events associated with TI. Limited evidence in the neonatal population has shown that VL also increases the success of neonatal intubation and has potential as a useful training tool (13). The purpose of this study was to compare the difference between direct laryngoscopy (DL) and VL in TI performed by trainees during neonatal resuscitation training and to explore the application value of VL.

## Methods

This was a prospective study aiming to investigate the use of VL and DL in neonatal tracheal intubation (NTI). The subjects of this study were the participants of the neonatal resuscitation simulation training camp (NRSC) from November 9–11th, 2018, to March 22–24th, 2019, and the midwives of the delivery room of the Shanghai First Maternity and Infant Hospital, China. The NRSC training course has been provided every other month beginning in 2016. The curriculum of the NRSC comprises five skill stations (including initial steps, positive-pressure ventilation, the six corrective Step, i.e., mask adjustment, reposition airway, suction mouth and nose, open mouth, pressure increase, alternative Airway (MRSOPA), TI, chest compressions (CPR) and UVC insertion on the first day and five rounds of simulation training on the second day and the morning of the third day. There were 24 trainees in each camp from all over the country, and they were mainly the medical staff involved in neonatal resuscitation in the delivery room, including pediatricians, nurses, obstetricians, and midwives. This study was approved by the Ethical Committee of the hospital, and consent from the participants was waived because patient safety was not involved in this study.

### Instruction for Intubation Procedure With VL or DL

Instructions for neonatal intubation were given as a demonstration to the trainee according to the NRP textbook (14). Briefly, the trainee stands by the head of the manikin and stabilizes the manikin's head with the right hand while the head is in the “sniffing” position. The manikin's mouth is gently opened; the laryngoscope blade is inserted into the right side of the mouth and slide over the right side of the tongue toward the midline. The tongue is gently pushed toward the left side of the mouth, and the blade is advanced until the tip lies just beyond the base of the tongue in the vallecula. The entire laryngoscope is lifted in the direction that the handle is pointing, moving the tongue out of the way to expose the glottis. The glottis appears at the very top of the view as facing the camera of the VL or the DL directly. Once the landmark is identified, the laryngoscope is held steady, the view of the vocal cords is maintained, and an assistant is asked to place the endotracheal tube in the right hand. The tube is inserted into the right side of the manikin's mouth. After insertion, the tube is directed into the hypopharynx and the tip is advanced toward the vocal cords. The tube is advanced until the vocal cords are positioned between the vocal cord guide lines. The tube is held securely against the manikin's upper palate. The laryngoscope is carefully removed without displacing the tube, and the endotracheal tube is secured.

**Figure 1:**
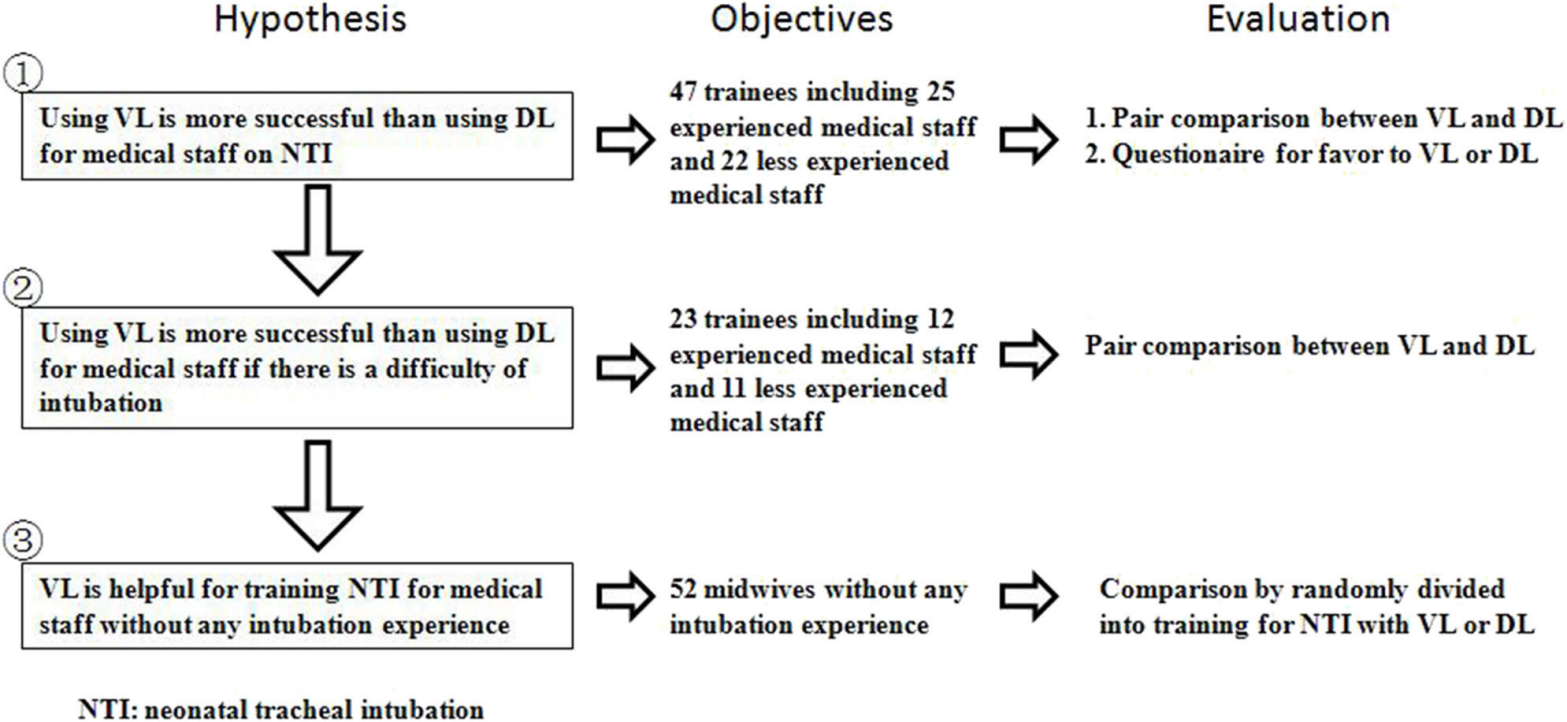
Research diagram.

The study was divided into three parts according to the difficulty of intubation (Figure 1) and the level of NTI experience. The level of NTI experience was identified by self-reporting many intubation experiences as experienced medical staff (EMS) or self-reporting few intubation experiences as less-experienced medical staff (LEMS).

**Figure 2:**
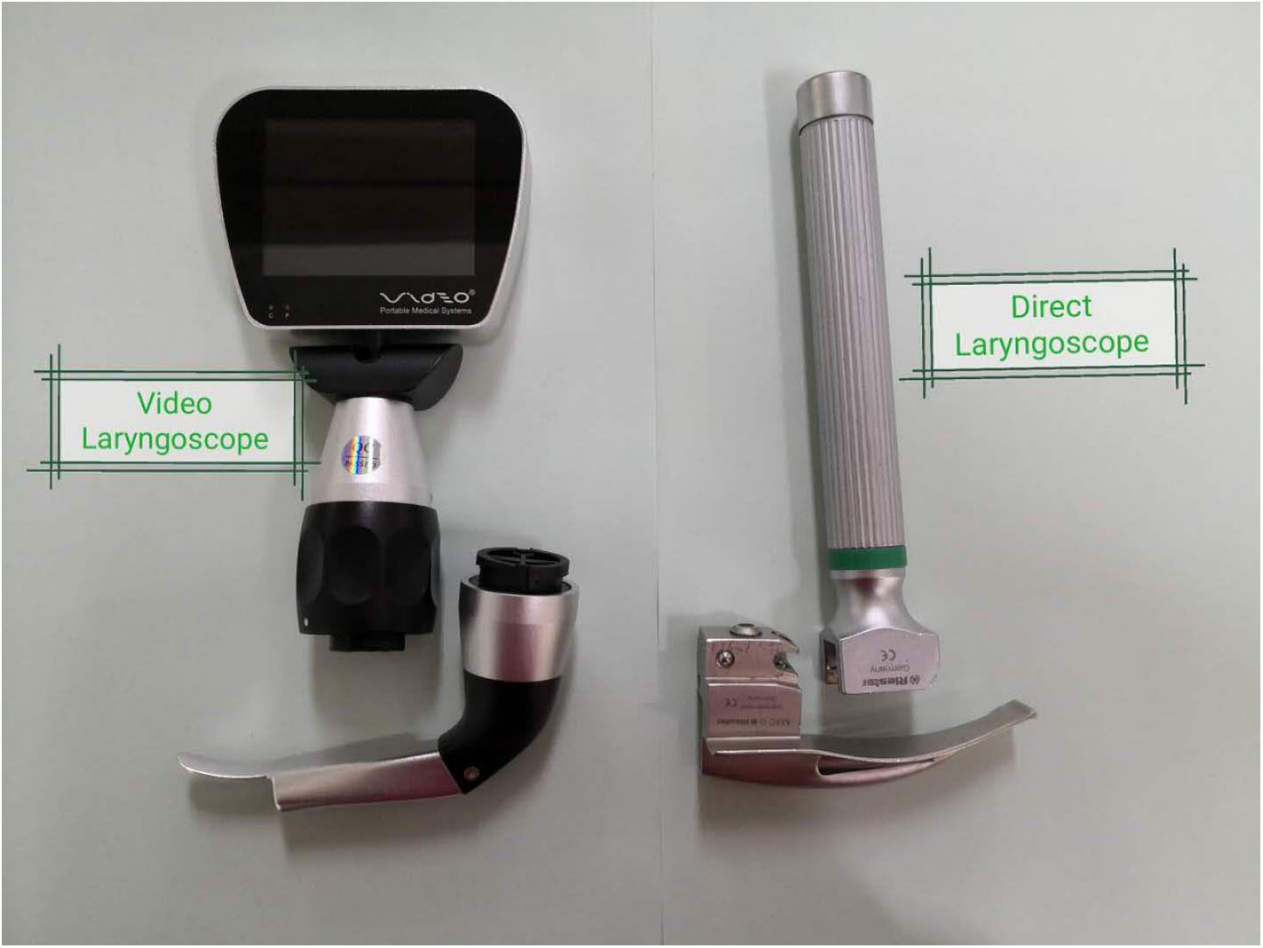
A direct laryngoscope and a video laryngoscopy with blade −1.

Part 1: Medical staff intubated a manikin under neonatal resuscitation. From November 9–11, 2018, to March 22–24, 2019 (during two consecutive training camps), the participants of the courses were randomly selected according to odd or even numbers of the group label number to test the difference between DL and VL in the neonatal resuscitation training by performing TI on a simulation manikin (SimNewB, Lardel) with a direct laryngoscope (Rlester, Germany) or a video laryngoscope (VDO-100c, VDO MEDICAL INC, Shanghai, China) (Figure 2) on the first and second day, respectively. The scenario was as follows: The infant was a non-vigorous, 40-wk singleton, and the amniotic fluid was clear. After positive-pressure ventilation (PPV) with bag and mask, there was insufficient manikin lung inflation, and the requirement for TI was identified. Participants were required to perform endotracheal intubation, with assistance from an instructor, within 30 s with a #1 blade 3.5-F tracheal tube. The other instructor used a stopwatch to monitor the time between when the blade was inserted into the mouth and when the blade was taken out of the mouth after the intubation was success or failure. After the intubation was completed, the trainee was required to ventilate through the resuscitation capsule 40–60 times/min and judge whether the intubation was successful by monitoring the air entry into the artificial chest by the computer of the manikin.

After 2 days of the TI training in both courses, participants were asked to complete a questionnaire on the differences between the direct laryngoscope and the video laryngoscope. There were six single-choice questions on the questionnaire, including questions regarding better handling, clearer visualization, easier visualization of the glottis, less postural demand, better confidence, and preferred method (Table 1).

**Table 1:** Comparison of intubation time and success rate between VL and DL.

**Items**	**Groups**	***n***	**VL**	**DL**	**T/t/*X*^**2**^**	***p***
NTI without intubation difficulty						
Intubation time	EMS	25	24.1 ± 7.2 s	18.1 ± 6.9 s	4.107	<0.001
	LEMS	22	25.6 ± 6.6 s	21.9 ± 7.0 s	1.884	0.073
Success rate	EMS	25	48.0% (12/25)	88.0% (22/25)^*^	7.445	0.006
	LEMS	22	68.2% (15/22)	40.9% (9/22)	2.292	0.130
NTI with the ongoing chest compressions						
Intubation time	EMS	12	23.9 ± 8.1 s	21.0 ± 7.3 s	1.243	0.24
	LEMS	11	22.8 ± 7.7 s	23.8 ± 6.6 s	0.395	0.701
Success rate	EMS	12	41.7% (5/12)	75.0% (9/12)	1.543	0.214
	LEMS	11	63.6% (7/11)	63.6% (7/11)	0	1
Midwives who were novices in NTI						
Intubation time	Before training	26	42.2 ± 25.8 s	30.5 ± 24.2 s	1.694	0.097
	After training	26	19.6 ± 9.0 s	28.0 ± 6.7 s	8.474	<0.001
Success rate	Before training	26	11.5 (3/26)	23.1 (6/26)		0.303
	After training	26	96.2% (25/26)	69.2% (18/26)		0.024

*DL, direct laryngoscopy; VL, video laryngoscopy; NTI, neonatal tracheal intubation; EMS, experienced medical staff; LEMS, less-experienced medical staff*.

* *p < 0.01 compared to that of LEMS on NTI success rate*.

**Figure 3:**
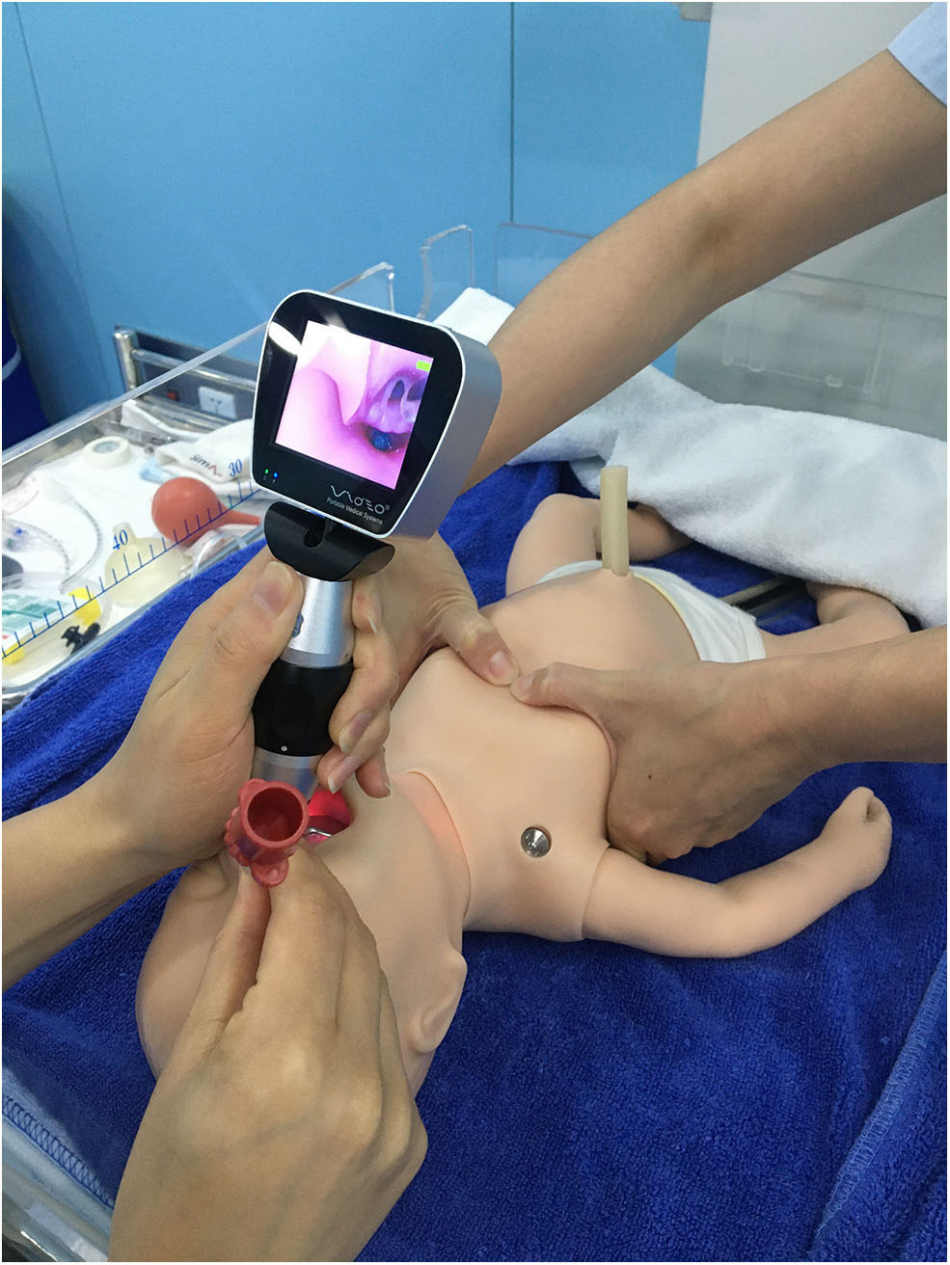
Neonatal tracheal intubation using a direct laryngoscope or a video laryngoscope when chest compression was continuous.

Part 2: Medical staff intubated a manikin with intubation difficulty. To test the difference in intubation between DL and VL under difficult conditions during neonatal resuscitation training, 24 participants from March 22–24, 2019, were asked to perform another intubation procedure under difficult intubation circumstances after the previous test. The scenario was as follows: The infant was a 40-week singlenton with fetal heart rate deceleration before delivery, and the amniotic fluid was clear. The heart rate was still 50 beats/min after 30 s of effective ventilation. It was decided to start chest compressions after TI. However, the endotracheal tube was observed to be dislodged after chest compressions, and the participants were asked to perform intubation with a #1 blade and 3.5-F tracheal tube within 30 s with ongoing chest compressions. One instructor was an assistant for tracheal intubation, and another participant in the same training group was required to perform external chest compressions 90 times per minute. The other instructor used a stopwatch to time the participant as in the previous test (Figure 3). After the intubation was completed, the trainees were required to carry out chest compressions and ventilation coordinated at a ratio of 3:1. The success of intubation was confirmed as in the previous test.

Part 3: Midwives without intubation experience performed NTI. To investigate the benefit of VL on the training of medical staff with no experience or training in NTI, endotracheal intubation training was set up in the delivery room of our hospital for midwives because they are unfamiliar with TI. The midwives were randomly divided into the DL and VL groups according to block randomization. TI was demonstrated by instructors using DL or VL with the midwives after the first attempt to intubate the manikin as previously (SimNewB, Lardel). The second attempt was made immediately after the trainees were instructed by the instructors using DL or VL according to the randomization. The measures of intubation were the same as in the previous tests.

### Statistical Analysis

The mean value ± standard deviation was used for measurement data, and the paired *t*-test was used for the comparison of normally distributed data between the two groups on NRSC course groups. The *t*-test was used for the comparison of data from the midwives' intubation training. The chi-square test was used for the count data. *P* < 0.05 was statistically significant.

## Results

There were 47 participants in these two training courses: 25 pediatricians or neonatologists, including 16 attending doctors with an average of 12.3 ± 6.4 years of experience, and 9 residents with an average of 4.6 ± 2.2 years of experience; three pediatric nurses with 6, 12, and 15 years of experience; 12 obstetricians, including 6 attending doctors with 18.8 ± 7.3 years of experience and 6 residents with 3.5 ± 3.0 years of experience; and 7 midwives with 11.6 ± 4.6 years of experience.

### Mean NTI Time and Success Rate Using VL or DL

Among the 47 trainees, there were 25 EMS who self-reported many intubation experiences and 22 LEMS who self-reported few intubation experiences. The mean intubation time of EMS using VL was longer than that using DL (24.1 ± 7.2 vs. 18.1 ± 6.9 s), whereas there was no significant difference between VL and DL for LEMS (25.6 ± 6.6 s vs. 21.9 ± 7.0 s). It took a much longer time for trainees to visualize the field of view through the camera when using VL than when using DL. EMS spent slightly less time on NTI than did LEMS using both VL and DL, but there were no statistically significant differences (both *p* > 0.05) (Table 1).

The success rate of EMS using VL was significantly lower (48.0%, 12/25) than that using DL (88.0%, 22/25). Interestingly, the success rate of LEMS using VL was higher than that using DL, while there was no statistical significance between VL and DL (68.2%, 15/22 vs. 40.9%, 9/22). Although the success rate of EMS was significantly higher than that of LEMS using DL (88%, 22/25 vs. 40.9%, 9/22, *x*^2^ = 9.555, *p* = 0.002), VL improved the success rate of LEMS compared to that of EMS (68.2%, 15/22 vs. 48.0%, 12/25, *x*^2^ = 1.212, *p* = 0.271).

### Questionnaire on the Use of VL or DL

Overall, the medical staff favored DL with respect to handling and confidence in intubation, whereas they favored VL with respect to clear visualization, visualization of the glottis and less postural demand (Table 2).

**Table 2:** Questionnaire on the use of video laryngoscopy (VL) or direct laryngoscopy (DL).

**Questions**	**DL**	**VL**	**Both**	***X*^**2**^**	***P*-value**
	***N* (%)**	***N* (%)**	***N* (%)**		
Better handling	26 (55.3)	15 (31.9)	6(12.8)	19.213	<0.001
Clear visualization	11 (23.4)	34 (72.3)	2(4.3)	52.149	<0.001
Easier to visualize the vocal cord	14 (29.8)	25 (53.2)	8(17.0)	14.234	<0.001
Less postural demand	12 (25.5)	23 (48.9)	12(25.5)	7.723	0.021
Better confidence	22 (46.8)	17 (36.2)	8(17.0)	9.638	0.008
Preferred method	19 (39.6)	16 (33.3)	13(27.1)	1.706	0.426

### Mean NTI Time and Success Rate Using VL or DL Intubation Difficulty

The scenario of ongoing chest compressions when NTI was required was designed to increase the difficulty of intubating the manikin in this study. The mean intubation time for EMS was slightly longer using VL than using DL (23.9 ± 8.1 vs. 21.0 ± 7.3 s), while the mean intubation time for LEMS was slightly shorter using VL than DL (22.8 ± 7.7 vs. 23.8±6.6 s). There were no differences in the mean NTI time using VL or DL between EMS and LEMS (*p* > 0.05) with ongoing chest compressions (Table 1).

For EMS, the success rate of intubation was lower using VL than using DL (41.7%, 5/12 vs. 75.0%, 9/12), whereas for LEMS, it was the same using VL and DL (Table 1). The success rate of EMS was lower than that of LEMS using VL, but it was slightly higher than that of LEMS by using DL (both *p* > 0.05).

### VL for NTI in Medical Staff Without Intubation Experience

A total of 52 midwives without any intubation experience participated in the study. They were randomly divided into two groups, the VL and DL groups, according to block randomization. The intubation time before training was 42.2 ± 25.8 s vs. 30.5 ± 24.2 s, respectively. VL took longer than DL, but there was no significant difference (*t* = 1.694, *P* = 0.097). The intubation success rate was 11.5% (3/26) vs. 23.1% (6/26) (*p* = 0.303).

After simulation training, the mean intubation time was 19.6 ± 9.0 s for VL vs. 28.0 ± 6.7 s for DL, and the NTI time was significantly shorter using VL than DL (*t* = 8.474, *P* < 0.001). The intubation success rate using VL was significantly higher (96.2%, 25/26) than that using DL (69.2%, 18/26; *p* = 0.024).

## Discussion

In neonatal resuscitation, TI is a critical and dangerous technique for medical staff to implement under the stressful circumstance of birth asphyxia (8). Although medical staff in the delivery room is trained in TI as a part of the resuscitation curriculum, it is difficult to maintain the skill, especially in small maternal centers, with very few opportunities for resuscitation (15). In addition to simulating and practicing on a manikin regularly to maintain the knowledge and skill of TI, VL might demonstrate the anatomy of the glottis more easily than DL for novices during intubation (16).

Neonatal intubation has been used since 1940 to establish an artificial airway in pediatric patients who need airway management (17). The exposure and identification of the anatomy of the larynx are the most important steps for successful TI using DL (18). An attendant in the delivery room responding to neonatal resuscitation needs much practice to improve the success rate of intubation (19). Failed intubation is associated with multiple intubation attempts, desaturation, airway trauma, and even brain damage if artificial airway establishment is delayed (20). Therefore, a video laryngoscope, or an indirect laryngoscope, was designed and introduced to improve the view of the glottis via a fiber optic scope and a video camera at the tip of the blade (Figure 1). It has been shown in adult studies that VL improved the view of the glottis and the success rate of intubation and reduced the incidence of complications associated with the intubation procedure (11). However, the conclusions among these studies were inconsistent (21, 22). There was no improvement in the intubation success rate in these studies, although it is an alternative to DL. In pediatric patients, VL also improved visualization of the glottis but not the success rate of intubation (12). However, intubation took longer using VL than DL in this population. The National Emergency Airway Registry for Children database in the US includes data on 8,875 TIs in this population; 10% of intubations were performed by VL. VL was more often used in cases of airway difficulty and failed respiratory ventilation. It reduced the risk of adverse events in TI but not the risk of severe adverse events or number of intubation attempts (23). In addition, the use of VL in emergency airway management has been increasing (24).

Neonatal resuscitation is a very stressful form of technology, and medical staff needs to make judgments and decisions in a very short timeframe and complete a series of operations to save lives. In this case, these processes will significantly affect the success rate of TI. Even for very experienced doctors, under stressful circumstances, the success rate of endotracheal intubation decreased from 88 to 69%, and the time required will be extended by 4 s in our study. If CPR is not expected to stop during intubation, the impact on intubation must be minimized. VL is an important technique to improve the visual field and minimize posture-related requirements. In our study, the success rate using VL was still significantly lower than that using the familiar DL technique, but the intubation time was not different, which shows that good visualization, and posture can improve the TI experience. Currently, data on VL and DL as intubation tools for neonatal subjects are limited and controversial. In neonatal clinical practice, an international registry study of intubation reported 2,607 neonatal intubations in the US, Singapore and Canada (25). The study showed that 21% of neonatal intubations in the NICU and 11% in the delivery room were implemented by VL and independently associated with a reduced rate of intubation-associated adverse events. VL provides a better view of the glottis but does not decrease the intubation time or improve the success rate. The use of VL might be beneficial and shorten the intubation time if a view of the glottis is difficult to achieve (26). Failed intubation has been shown to be highly associated with anatomical visualization (18). Another training study using a simulation also proved that VL increased the success of intubation performed by pediatric residents (27). However, a meta-analysis of these trials demonstrated that while VL improved the success rate of neonatal intubation, the evidence was not strong (13). The intubation success rate was lower and the time requirement was longer using VL than using DL for the EMS in our training courses. Medical staff with a high level of experience with neonatal intubation prefers to use DL, since they feel more confident and comfortable with handling a familiar tool during an intubation practice in our training course (28). For resuscitators experienced with DL, it may take longer to finish the procedure, but with little difference in the success rates (29).

The resuscitator may sometimes perform intubation with ongoing chest compressions. Although few studies have reported that chest compressions may not interrupt the intubation process (30, 31), which is similar to the results of our study, it is important to reduce the risk of injury and relieve the stress of the intubator when continuous chest compressions are necessary. VL may improve the intubation time by providing a good view of the glottis compared with DL under the circumstances of uninterrupted chest compressions (32). These studies, including ours, reflect the performance of resuscitators or trainees in intubating manikins. Therefore, an observational study or clinical trial of VL vs. DL on this topic is warranted.

The trainees of the NRSC reported on our questionnaire that VL facilitates easier visualization of the anatomy and imposes less of a postural requirement on the medical staff, even with the first use of VL. One-third of the trainees with intubation experience favored its use as a result of the minor adaptation of visualization for intubation. The blade of the video laryngoscope may be designed to minimize the possibility of intubation-associated injury since the vocal cord do not have to be visualized for intubation (33). This method may help operators predict the difficulty of intubation (34). On the other hand, in efforts to train medical students with no intubation experience, VL improved the success rate of intubation in neonatal manikins, and this skill was transferrable to DL (35). As a training tool, VL provides feedback to learners. It allows learners to adapt the skill and translate it into clinical practice (36). VL could shorten the intubation time and improve the success rate significantly in training junior intubators or medical students. In our study, the novice providers tended to learn intubation skills more quickly using VL than using DL and translated them into clinical techniques, while more experienced providers performed better with DL (28).

There are some limitations to this study. First, it was a manikin study and may not fully reflect the clinical environment or the difficulty of neonatal intubation. The airway of the manikin is visually similar to that of a human newborn (Figure 1). We tried to minimize these effects in the simulation training course. The manikin training and the observations of this study can be translated into clinical practice. Second, the experience of the trainees was self–reported, which may have introduced bias. However, it was difficult to measure the intubation skill level of the trainees. Last, VL is a new intubation tool that many trainees were using for the first time, which may affect their performance intubating using VL. The trainees were given an opportunity to practice intubation on the same manikin using DL and VL at a skill station on the first day of the simulation course.

In conclusion, for medical staff with intubation experience using DL, VL did not improve the intubation success rate or the mean time. The intubation success rate using VL or DL was slightly affected by ongoing chest compressions. For midwives without any TI experience, VL training can significantly improve the intubation success rate, and shorten the intubation time.

## Data Availability Statement

All datasets generated for this study are included in the article/supplementary material.

## Ethics Statement

This study was approved by the Ethics Committee of Tongji University School of Medicine. The requirement for written informed consent was waived by the ethics committee.

## Author Contributions

MZ and JQ-L analyzed the data and drafted the manuscript. XX, ML, and SW collected the data. ZL and JQ-L designed the study. All authors contributed to the article and approved the submitted version.

## Conflict of Interest

The authors declare that the research was conducted in the absence of any commercial or financial relationships that could be construed as a potential conflict of interest.

## Abbreviations

VL: video laryngoscopy
NTI: neonatal tracheal intubation
DL: direct laryngoscopy
NRSC: neonatal resuscitation simulation training camp
CPR: chest compression
UVC: umbilical vein catheters
MRSOPA: mask adjustment, reposition airway, suction mouth and nose, open mouth, pressure increase alternative Airway.
